# Telehealth for Integrated Cardiovascular and Diabetes Management: A Scoping Review

**DOI:** 10.1155/jdr/1093671

**Published:** 2025-12-02

**Authors:** Maria Dulce Estêvão, Mónica Teotónio Fernandes, Ana Luísa De Sousa-Coelho, Margarida Espírito-Santo, Tânia Nascimento

**Affiliations:** ^1^School of Health-University of Algarve (ESSUAlg), Faro, Portugal; ^2^Algarve Biomedical Center Research Institute (ABC-Ri), Faro, Portugal; ^3^Faculty of Sciences and Technology-University of Algarve (FCT-UAlg), Faro, Portugal

## Abstract

Cardiovascular disease (CVD) and diabetes mellitus represent major global health challenges, frequently co-occurring and mutually exacerbating. Telehealth interventions offer a promising approach for their management, with potential to improve patient outcomes, enhance access to care, and increase cost-effectiveness. This review synthesized existing evidence from randomized controlled trials (RCTs) and observational studies to evaluate the effectiveness of telehealth interventions for the management of diabetes, focusing on CVD risk, and to identify critical research gaps. A systematic literature search was conducted across major databases (PubMed, Web of Science, and Scopus) to identify studies meeting predefined eligibility criteria, considering digital tools for remote monitoring, consultation, education, and medication management. After the screening of 3041 articles, six studies met the inclusion criteria. Telehealth interventions utilized a range of digital health tools, including mobile applications, artificial intelligence–powered clinical decision aids, electronic consultations, and integrated remote monitoring platforms. Although direct assessment of composite cardiovascular risk was largely absent, the included studies reported several clinical parameters associated with cardiovascular health, namely, blood pressure, lipid profile, and glycated hemoglobin. Telehealth interventions implemented for individuals with Type 2 diabetes mellitus demonstrated promising potential in improving glycemic control and supporting self-management. However, their effectiveness in managing broader cardiovascular risk factors remains less clear due to inconsistent reporting and heterogeneous intervention designs.

## 1. Introduction

Cardiovascular diseases (CVDs) refer to a broad group of different medical conditions, including arrhythmias, heart failure, coronary heart disease, cerebrovascular disease, and peripheral arterial disease, among others, with the commonality of being disorders that affect the blood vessels and the heart [1]. According to the World Health Organization, CVDs are a leading cause of death due to noncommunicable diseases, mainly owing to the occurrence of stroke, acute myocardial infarction, or heart failure [2]. In low- and middle-income countries, insufficient early diagnosis and correct management lead to excessive CVD-related premature deaths [3]. Additionally, air pollution has also been identified as a factor contributing to the increased risk of CVD events, both at the chronic and acute levels of exposure [4]. The risk of CVD is elevated by numerous conditions, including overweight or obesity, insulin resistance or Type 2 diabetes mellitus (T2DM), dyslipidemias, hypertension, and physical inactivity [5], with aging and genetic factors also playing a significant role [6, 7].

Primary healthcare programs play a crucial role in the early identification and reversal of modifiable cardiovascular risk factors (e.g., tobacco cessation), as well as in implementing effective management interventions, including appropriate pharmacological therapies [8]. For instance, achieving adequate blood pressure control contributes to the reduction of CVD risk in elderly patients with diabetes [9]. Furthermore, from an economic perspective, early detection strategies for CVD have proven largely cost-effective, significantly reducing CVD-related expenses [10].

Diabetes refers to a chronic state of hyperglycemia, caused by a combination of alterations in carbohydrate metabolism, namely, deficient glucose uptake and utilization by skeletal muscle cells and adipocytes, and inappropriate hepatic glucose production [11]. Recent epidemiological data on diabetes mellitus highlights a rapidly increasing global burden of the disease. This reflects increased prevalence but also increased levels of undiagnosed individuals and regional disparities. As for CVD, diabetes has the highest prevalence in low- and middle-income countries [12, 13]. Moreover, many of the risk factors associated with diabetes, such as obesity, physical inactivity, unhealthy diet, dyslipidemia, hypertension, and smoking, are also prominent contributors to CVD [14]. This strong overlap means that CVD is the leading cause of mortality in both Type 1 diabetes mellitus (T1DM) and T2DM [5].

As previously highlighted, there is a converging pathophysiology of CVD and diabetes. Not only do both diseases share common risk factors, but CVD signs and symptoms also show similarities with those in patients with T2DM, namely, shortness of breath and reduced exercise capacity or tolerance [15, 16]. The Framingham study had a massive importance in the field of cardiovascular medicine, as it was the first to systematically identify the CVD risk factors and to develop risk prediction models and useful scores, currently used for risk assessment and prevention [17]. Early reports showed that having diabetes diagnosis increased the risk of CVD in both sexes [18]. Because of the strong bidirectional relationship between diabetes and CVD, it is, therefore, essential that patients with diabetes are screened for CVD and that screening for T2DM is performed in all patients with CVD [8].

Managing chronic diseases often poses many challenges to patients, namely, a lack of therapeutic adherence. The American Diabetes Association recommends that, based on the patients' comorbidities, antihyperglycemic agents that also benefit cardiovascular and kidney outcomes should be selected [19], namely, Glucagon-Like Peptide 1 receptor agonists and Sodium–Glucose Cotransporter-2 inhibitors [5]. In addition, it is currently advocated that patients should take advantage of novel diabetes technology, such as continuous glucose monitoring and insulin infusion devices [20]. Nevertheless, it is required that patients not only receive the initial training but also have access to ongoing education and evaluation of its use, either personally or remotely.

Overall, most patients may control their diseases by correctly taking the prescribed medication and following the recommendations on lifestyle habits, including regular physical activity and balanced diets. However, this commitment can be undermined by several factors, leading to a lack of compliance. Specifically, patients may not have access to routine consultations when needed, and the symptoms of uncontrolled diseases are often nonspecific and remain silent for long periods. Patients may, however, use their close relationship with community pharmacists [21], who are more accessible to any individual and may dilute this potential lack of follow-up. Nevertheless, considering how individuals, at almost any age, currently have access to smartphones and computers with Internet access, healthcare digital tools may contribute to improving both cardiovascular and diabetes management.

Telehealth refers to the delivery of healthcare services using digital resources, such as electronic information and communication technologies to support long-distance clinical healthcare, including diagnosis, treatment, and prevention of diseases and injuries, patient and professional health–related education, public health, and health administration [22–24]. Therefore, unlike telemedicine, a term used interchangeably that specifically refers to the provision of clinical services remotely, telehealth is broader in scope, integrating both clinical and nonclinical components of health service delivery. Indeed, it encompasses a wide range of services and modalities, including real-time (synchronous) or delayed (asynchronous) interactions through video conferencing, remote monitoring tools, mobile health (mHealth) applications, electronic prescribing, or other digital platforms [22, 24–27]. Telehealth has emerged as a promising paradigm to address many limitations of traditional care models as it offers the potential to enhance access to care, improve convenience for patients and providers, facilitate continuous monitoring and timely intervention, promote patient self-efficacy, and potentially reduce healthcare burden [25, 28].

Although the first large-scale telemedicine programs were implemented back in the 1970s and facilitated over time due to the advancement of digital technologies, the COVID-19 pandemic served as a major inflection point for the rapid adoption and normalization of telehealth across healthcare systems worldwide. With in-person visits restricted, digital health technologies became essential for maintaining continuity of care, especially for patients with chronic conditions [29, 30]. Concurrently, advances in artificial intelligence (AI) significantly enhanced the capabilities of telehealth platforms, enabling automated triage, remote monitoring, personalized treatment recommendations, and improved clinical decision-making [31, 32]. This convergence of pandemic-driven necessity and AI innovation accelerated the shift toward hybrid and data-driven models of care, laying the groundwork for a more accessible, efficient, and scalable healthcare infrastructure.

As mentioned before, among the most pressing challenges in contemporary chronic disease management is the dual burden of diabetes mellitus and CVD. Traditional models of care and telehealth approaches, which often isolate diabetes management from cardiovascular risk reduction strategies, fail to address the intertwined pathophysiological and behavioral components of these conditions [33–36]. Therefore, given the intricate link between CVD and diabetes and the complexities of their concurrent management, telehealth modalities are particularly well suited to support integrated care approaches. As examples, telehealth interventions can facilitate simultaneous monitoring of glycemic control, blood pressure, body weight, and other relevant parameters; enable coordinated communication between primary care physicians, cardiologists, endocrinologists, and other specialists; deliver tailored educational content; and provide ongoing behavioral support [33, 37–39]. Such integrated telehealth strategies hold the potential to provide a more holistic, patient-centered approach compared to siloed care pathways, potentially leading to improved clinical outcomes for this high-risk population.

Further supporting a combined approach to diabetes and CVD through telehealth, recent applications extend beyond conventional remote consultations to include AI-powered decision support systems, applicable to biosensors for continuous monitoring, mobile software applications (apps) for self-management of chronic diseases, and digital platforms that integrate patient data across different specialties [37, 40, 41]. These AI-enhanced tools further augment the ability of telehealth systems to deliver proactive, personalized care by anticipating clinical needs, detecting early warning signs, and optimizing care coordination across multidisciplinary teams.

However, despite this promise, the evidence base remains fragmented. Existing reviews typically examine either diabetes or CVD management in isolation or focus broadly on telehealth without specifically addressing AI-based applications that target cardiovascular risk outcomes [42–45]. To our knowledge, no prior synthesis has explicitly evaluated telehealth interventions that leverage AI to support the integrated management of diabetes alongside cardiovascular risk factors.

Therefore, the goal of this review is to address this critical gap by identifying, evaluating, and synthesizing evidence from RCTs and observational studies that investigate telehealth interventions for adults with diabetes, with a particular focus on AI-enabled tools and their impact on cardiovascular risk outcomes (HbA1c, blood pressure, and lipid markers). By restricting our scope to studies that report both diabetes and cardiovascular-related outcomes, this review provides a novel and timely contribution to the literature, highlighting the current effectiveness of AI-integrated telehealth strategies and identifying gaps to guide future research, policies, and clinical practice.

## 2. Materials and Methods

The methodology used follows a previously registered protocol (https://osf.io/gbxdy/?view_only=7a0734e862fa463dbb1c151c5b83cdb1), despite some adjustments regarding the data extracted from the included studies.

### 2.1. Search Strategy

This review complies with the PRISMA Extension for Scoping Reviews (PRISMA-ScR) guidelines [46] to ensure methodological transparency and reproducibility (Supporting Information S1). The research strategy and eligibility criteria were structured using the PIO (population, intervention, and outcome) framework, which is a variation of PICO suitable for intervention-focused reviews, ensuring clarity and focus on the intervention of interest (telehealth). The search strategy was developed using the 2DSearch platform (https://www.2dsearch.com/).

The final strings obtained were adjusted for the different databases used and reviewed by all team members. All published studies were retrieved from MEDLINE/PubMed (National Center for Biotechnology Information, NCBI), Scopus (Elsevier), and databases accessible via the Web of Science from inception to April 4, 2025. No limits or filters were applied. Supporting Information S2 shows the search string used in each database and the respective number of retrieved articles. No search for grey literature, conference proceedings/abstracts, and preprints was carried out.

Duplicated references were identified through Rayyan [47] and manually revised by one reviewer (MDE).

### 2.2. Eligibility Criteria

Studies were selected according to the following criteria:

Inclusion criteria were as follows:

- Study designs: Clinical trials (randomized and nonrandomized) and observational studies (e.g., cohort and case-control) in which the design of the study reported the outcome data of interest longitudinally.
- Participants: Individuals diagnosed with T1DM or T2DM and/or caregivers involved in their diabetes management.
- Intervention: Studies evaluating interventions that utilize multimedia resources (e.g., video or image sharing), dedicated digital platforms, or remote monitoring based on devices/applications, allowing data exchange or synchronous or asynchronous visual or textual interaction, and AI-based tools aimed at the management of diabetes, specifically addressing or assessing CVD risk factors.
- Outcomes: Studies reporting both baseline and end-of-study data for at least one of the following cardiovascular risk outcomes:
  - Glycated hemoglobin (HbA1c)
  - Fasting blood glucose (FBG)
  - Blood pressure (systolic blood pressure [SBP] and/or diastolic blood pressure [DBP])
  - Lipid profile markers (triglyceride [TG], total cholesterol [TC], low-density lipoprotein cholesterol [LDL-c], and/or high-density lipoprotein cholesterol [HDL-c])
- Context: No limitations imposed based on language of publication, geographic location of the study, or participant sex, race/ethnicity, or age.

Exclusion criteria were as follows:

- Publication type: Secondary research (e.g., narrative reviews, systematic reviews, and meta-analyses), study protocols, conference abstracts or proceedings, editorials, letters, opinion pieces, preprints, and any publication type not reporting primary data, namely, cross-sectional and qualitative studies, as they do not provide the pre- and postintervention data necessary for evaluating effectiveness.
- Participant population:
  - Studies focused exclusively on participants with gestational diabetes mellitus (GDM) or where it is not possible to obtain separate data on patients with T1DM and/or T2DM.
  - Patient/caregiver-centered studies that use other disease management processes instead of digital- or AI-based tools.
- Intervention focus: Studies evaluating digital- or AI-based tools for diabetes management in which the focus or outcomes reported relate exclusively to non-CVD risks (e.g., diabetic foot complications, retinopathy, nephropathy, and depression) or which do not report any of the required CVD risk outcomes listed in the inclusion criteria.
- Outcome reporting: Studies that do not report quantitative data for at least one of the specified CVD risk outcomes or studies lacking data corresponding to the beginning of the observation/intervention period (baseline data).
- Accessibility: Studies for which it is not possible to obtain the full text of the manuscript or to translate it reliably into English (or into the working languages of the review team).

### 2.3. Screening

Once deduplication was complete, the remaining articles were screened by pairs of independent reviewers (MDE, TN, or MES) according to predefined eligibility criteria. This evaluation proceeded in two phases: an initial screening of title and abstract, followed by a full-text review of selected articles. Due to the nature of this scoping review, the quality of included studies was not systematically assessed (e.g., risk of bias or GRADE assessment was not performed).

### 2.4. Data Extraction

Data extraction was carried out by one reviewer (MDE) and then checked by a second member of the review team (TN). In case of conflict, the final decision was reached by consensus. An Excel extraction form was developed specifically for this purpose.

Extracted data includes (1) article identification and study characterization (name of the first author, title, year of publication, country where the study was carried out [in its absence, the country of the affiliation of the first author was considered], journal, study design, funding sources, and trial ID, when applicable), (2) participants (number, mean age and standard deviation [SD] or median, interquartile range [IQR], sex, race/ethnicity, health condition [T1DM or T2DM], and comorbidities), (3) digital tool used (designation, type, main features, and other relevant information, (4) intervention (duration, randomization process [when applicable], reported outcomes of interest [according to the inclusion criteria] at baseline and endpoint, overall improvement of self-management, cardiovascular risk assessment, and other relevant reported outcomes), and (5) information reported by the authors (main conclusions and strengths and limitations).

Data are presented in summary tables which support the results description.

## 3. Results

**Figure 1:**
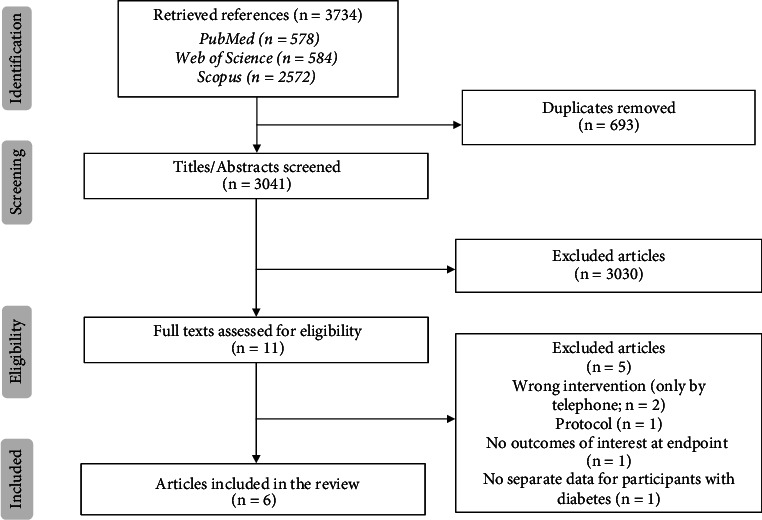
PRISMA flowchart.

After the initial search for studies evaluating the use of telehealth for cardiovascular and diabetes management, a total of 3734 articles were identified. After deduplication, 3041 unique articles were screened based on their title and abstract, leading to the retrieval of 11 articles for full-text eligibility assessment. Of these, six met the inclusion criteria. This number reflects the highly focused nature of the review's eligibility criteria, which specifically targeted studies addressing app-based telehealth for the integrated management of both CVD and diabetes. The five excluded articles were rejected for the following reasons: intervention conducted exclusively by telephone (*n* = 2), study protocol (*n* = 1), absence of data on the outcomes of interest at the intervention endpoint (*n* = 1), and mixed data that did not allow for separation of analysis of participants with diabetes (*n* = 1). The complete study selection process is illustrated in the PRISMA flow diagram (Figure 1).

### 3.1. Characteristics of Included Studies

The six included studies were published between 2021 and 2025. Geographically, the studies were conducted in the United States (*n* = 2) [48, 49], China (*n* = 1) [50], Singapore (*n* = 1) [51], Italy (*n* = 1) [52], and Belgium (*n* = 1) [53]. Study design comprised four observational or nonrandomized trials [48, 50, 52, 53] and two randomized controlled trials (RCTs) [49, 51]. All studies received funding, with two reporting support from industry sources [48, 53]. Across all studies, a total of 201,868 participants with T2DM were included. Sample size ranged from 66 [53] to 199,431 [50] participants, and the mean age of participants varied from approximately 54 to 67 years. While all studies included both sexes, only three provided data on race or ethnicity [48, 49, 51]. Participant comorbidities were reported in four studies [48–50, 53]. A detailed summary of these study characteristics is provided in Table 1.

### 3.2. Characterization of the Telehealth Interventions

The interventions were delivered via diverse digital platforms. These primarily consisted of mobile applications, some of which with integrated AI support [48, 50], and electronic consultations [49]. The platforms encompassed a wide range of features, such as patient management systems, AI-driven insulin titration algorithms, remote collection of clinical parameters, clinical decision support tools, therapeutic education modules, and medication adherence monitoring (Table 2).

The duration of the interventions ranged from 3 to 24 months. Regarding study design, randomization procedures were described for the two RCTs [49, 51]. In fact, Finkelstein et al. included three groups: the usual care (UC) group (control), the diabetes management program (DMP) group, and a third group that, in addition to the DMP, received rewards for healthy behaviors (DMP+) [51]. De Luca et al. [52], in a non-RCT exploratory trial, employed a cluster allocation design, where randomization occurred at the level of the healthcare center instead of the individual participant. A comprehensive summary of all intervention characteristics is provided in Table 2.

### 3.3. Cardiovascular Risk–Related Outcomes

The analysis focused on three categories of cardiovascular risk markers: glycemic control, lipid profile, and blood pressure. There was considerable heterogeneity in outcome reporting across the six studies. Frequently, parameters measured at baseline were not reassessed or reported at the study endpoint, limiting the ability to determine the effects of the intervention. All findings are summarized in Table 3.

#### 3.3.1. Glycemic Control

HbA1c, a measure of long-term glycemic control, was the most common outcome reported. Four studies showed a statistically significant reduction in HbA1c compared to baseline values after the intervention [48, 50–52]. Conversely, two studies found no significant changes in HbA1c levels between baseline and follow-up in the intervention group [49, 53]. Among observational and quasiexperimental studies, three show statistically significant differences compared to baseline [48, 50, 51]. However, only one experimental study shows significant differences when comparing the control and intervention groups. Finkelstein et al. [51] showed significant differences between the DMP+ group and the UC group (*p* = 0.015). Although a decrease in HbA1c was reported in the DMP group, it was greater and more significant in the DMP+ group. De Luca et al. [52] demonstrated differences in HbA1c compared to baseline but without significance between the study groups.

#### 3.3.2. Blood Pressure (SBP and DBP)

Four studies reported blood pressure outcomes, although complete baseline and endpoint data were available for only three of them [51–53]. A significant reduction in both SBP and DBP was only evident in the study by De Luca et al. [52]. This effect was statistically significant for both the within-intervention group change (paired analysis from baseline to endpoint) and the between-group difference when compared to controls at the 8-month follow-up (endpoint). In contrast, the control group did not exhibit significant changes in BP from baseline to the end of the study (Table 3).

#### 3.3.3. Lipid Profile

The lipid profile was the least frequently reported outcome, with endpoint data available in only two studies [52, 53]. In one of these, the intervention led to a significant within-group decrease in TC and LDL-c levels only in the mobile application group but not in the control group [52]. Indeed, these parameters were significantly reduced in the intervention group when compared to the control group at the end of the study. By contrast, the study by Lallemand and colleagues reported no significant changes in lipid parameters from baseline to the endpoint [53].

### 3.4. Additional Health and Patient-Reported Outcomes

In addition to cardiovascular risk markers, data on broader health outcomes, patient-reported outcomes, and healthcare professional feedback were also analyzed (Table 3). Positive effects on general disease management were reported in three studies [50–52]. Two studies noted improvements in other cardiovascular risk–related outcomes, namely, reductions in body weight and waist circumference [52, 53]. Additionally, two other studies reported a lower incidence of hypoglycemia in the intervention groups [48, 50].

High levels of patient satisfaction with digital interventions were reported in four studies [48, 50, 52, 53]. By contrast, one study reported no significant changes in overall quality of life and observed a statistically significant decline in both sleep quality and work productivity among participants in the intervention group [51]. Finally, two studies reported positive engagement and feedback from the healthcare professionals involved [49, 53].

## 4. Discussion

In this review, the effectiveness of telehealth interventions for diabetes management with a focus on their effect on cardiovascular risk was evaluated by synthesizing findings from six studies that met predefined inclusion criteria. Although this review adhered to the principles of scoping review methodology and did not encompass quantitative synthesis, methodological rigor was ensured through the implementation of a predefined protocol, incorporating dual-review verification and consensus procedures to mitigate potential biases in data extraction. The included studies were geographically diverse and spanned a range of research designs, digital tools, and outcome metrics, underscoring the global relevance and growing interest in telehealth as a care delivery model.

### 4.1. Overview of Included Studies and Participants

The six studies included in this review, published between 2021 and 2025, were conducted across diverse healthcare systems in the United States, Europe, and Asia. The sample sizes varied substantially, from as few as 66 to nearly 200,000 participants, reflecting a spectrum of study designs ranging from small-scale pilot interventions to national-level digital health programs. All studies focused on individuals with T2DM and generally reported similar demographic characteristics with respect to age and sex distribution, with the exception of the study by De Luca et al., with a notably male-dominated sample: 70% in the control group and 83% in the intervention group [52].

Although most studies were relatively short in their duration, they experienced substantial participant attrition, resulting in a smaller number of individuals reaching the study endpoint. Furthermore, the age range of participants was relatively narrow, which may limit the applicability of findings across age groups. Since the average age of diagnosis for T2DM is around 50 years, it is to be expected that most studies including patients with Type 2 diabetes will have samples with higher ages [54], which could bias the results, as age can be a barrier to the use of mHealth apps [55, 56].

Additionally, three of the six studies [48, 50, 53] did not include a control group unexposed to the telehealth intervention, further weakening the ability to draw causal inferences and limiting the generalizability of their findings.

Given that digital health literacy and socioeconomic status are likely to influence both the uptake and effectiveness of telehealth tools for diabetes self-management, as well as the underlying cardiovascular risk, it is essential that future studies consider these variables when defining eligibility criteria and in the analysis and reporting of outcomes [55, 57]. Incorporating these factors will enhance the relevance, equity, and translational potential of future telehealth interventions for chronic disease management.

### 4.2. Nature and Features of Telehealth Interventions

The interventions utilized a spectrum of digital health tools, including mobile applications, AI-powered clinical decision aids, electronic consultations, and integrated remote monitoring platforms. These tools often combine multiple functionalities such as glycemic monitoring, medication adherence tracking, automated insulin titration, and patient education. Such integration reflects a growing recognition of the need for comprehensive and multifaceted telehealth solutions to manage complex chronic conditions like diabetes, especially when coupled with cardiovascular risk factors.

AI-enhanced systems, as demonstrated in the included studies by Warren et al. [48] and Li et al. [50], illustrate the advanced potential of digital health technologies in optimizing insulin titration and delivering real-time clinical decision support. These tools have the potential to reduce the burden on both patients and healthcare providers by automating key aspects of diabetes management and enabling more timely, personalized care. However, the considerable variability in technological sophistication, implementation strategies, and levels of system integration underscores the need for greater standardization. Establishing common frameworks and reporting practices is essential to enhance cross-study comparability and to facilitate broader scalability and integration into routine clinical practice.

So far, despite the increased development of AI models and tools for several different tasks, their use in telehealth and, specifically, to support disease self-management is still scarce [58, 59]. It is important to make sure that once these tools are available, their implementation and evaluation will be done by carrying out well-designed trials, which allow an understanding of their benefits and risks.

### 4.3. Effectiveness of Interventions on Cardiovascular Risk–Related Outcomes

The present review gathered the available evidence on the use of digital tools for assessing diabetes management, focusing on cardiovascular risk, but the results show that this parameter was not directly determined. Instead, several clinical parameters associated with cardiovascular health, such as glycemic control, blood pressure, and lipid profile, were measured. Although the objective of assessing glycemic control may not be directly related to cardiovascular risk control in most of the studies included in this review, it is known that maintaining glycemia under controlled values is associated with a lower cardiovascular risk, reducing the incidence of nonfatal acute myocardial infarction [60, 61].

Other conditions, such as dyslipidemia or hypertension, are also known cardiovascular risk factors that are important in patients with diabetes [62, 63]. Intensive blood pressure control reduces the risk of stroke by 29% and the risk of heart failure by 31% in patients with diabetes [64], although blood pressure targets should be individualized, and evidence suggests that such rigorous control should not be applied in the elderly [63]. Similarly, the control of dyslipidemia (cholesterol and/or TGs) is an important factor in reducing cardiovascular risk in these patients [65, 66].

Glycemic control was the most consistently reported outcome. Four studies demonstrated statistically significant reductions in HbA1c levels as compared to baseline [48, 50–52], but only one of the three studies showed statistically significant differences between the intervention and control groups, supporting that digital interventions can enhance self-management and improve glycemic control [51]. However, it is important to note that in this study, one of the intervention groups received rewards, and those who received more rewards showed a greater reduction in HbA1c (*p* = 0.005). The two other studies that also had a control group but did not report such benefits [49, 52] may reflect differences in intervention intensity or participant adherence, for example. These mixed results indicate that while digital tools can be effective, their success is context-dependent and likely influenced by user engagement and the level of integration with routine care.

Blood pressure outcomes were less frequently and consistently reported. Only one study demonstrated significant reductions in both SBP and DBP between the intervention and control groups [52]. The limited and heterogeneous reporting makes it difficult to draw robust conclusions regarding the impact of telehealth on blood pressure control in this population. Moreover, lipid profile outcomes were also underreported. Among the two studies that provided endpoint lipid data, only one found a slight impact of a digital solution for managing T2DM on the blood lipid profile [52]. However, both parameters are extremely important for assessing cardiovascular risk and are used as essential variables in several scores such as SCORE2-Diabetes [67, 68]. This may be one reason why we have not found any studies that evaluated the direct impact on reducing cardiovascular risk [69]. Nonetheless, the observed improvement in glycemic control aligns with findings from previous studies [45, 70–74].

Interestingly, although biometric measures like body weight and abdominal circumference are not typically used to categorize cardiovascular risk as low, intermediate, or high for near-term events, reducing overweight or obesity can play a significant role in improving dyslipidemia and hypertension [75, 76]. Indeed, while most do not use them [77, 78], the UK-based QRISK3 score uses the patient's BMI in its calculation [79]. Nevertheless, only two of the included studies reported such alterations after the intervention (waist circumference and/or body weight) [52, 53], which may have contributed to the reduction in cardiovascular risk. This paucity of data underscores a significant gap in the current evidence base and emphasizes the need for more robust study designs, particularly those with longer follow-up periods and comprehensive outcome measures, to effectively assess whether telehealth can deliver fully integrated cardiovascular and metabolic benefits for individuals with diabetes. Importantly, one of the studies did report improvements in both blood pressure and lipid levels [52], which are well-established cardiovascular risk factors. These findings are consistent with prior research demonstrating that integrated interventions, particularly those incorporating lifestyle modifications and adherence to therapies, can positively influence these parameters [80–83]. Reducing cardiovascular risk in patients with diabetes is a challenge in clinical practice [5, 19]. The strategies used by the studies included in this review were much more educational or focused on controlling various clinical parameters, but none presented a digital solution that aggregated all cardiovascular risk parameters (blood glucose, HbA1c, blood pressure, cholesterol, and triglyceride levels, among others) in patients with diabetes and analyzed them together to obtain a clinical decision support tool for cardiovascular risk. Other reviews also describe telemedicine as a supportive strategy in controlling cardiovascular risk in patients with diabetes [42, 84]. The use of AI, on the other hand, is more recent and presents several challenges but also opportunities in the approach to patients with diabetes [85]. While AI seems to be growing in terms of predicting the risk of developing diabetes or complications, or even in systems to support therapy optimization, there are still some challenges in predicting cardiovascular risk [86].

### 4.4. Strengths and Limitations of the Current Evidence

The strength of the current evidence lies in the use of real-world data, diverse settings, and inclusion of both RCTs and observational studies. However, several limitations affect the interpretability and generalizability of findings. First, the heterogeneity in study design, intervention components, and outcome measurement precludes meta-analysis and complicates direct comparisons. Second, incomplete outcome reporting, especially regarding cardiovascular risk markers beyond glycemic control, restricts the ability to assess the full impact of these interventions. Third, many studies failed to report long-term outcomes, raising concerns about the sustainability of observed benefits.

Moreover, although many interventions focused on self-monitoring and management, few explicitly incorporated structured behavioral or lifestyle change components such as dietary counseling or physical activity promotion. These are critical for holistic cardiovascular and diabetes risk reduction. Another important shortcoming was the limited reporting of disaggregated outcomes by sociodemographic variables such as age, race/ethnicity, or socioeconomic status, factors that are essential for understanding the equity and accessibility of digital health interventions. Future research should strive, for example, to include more diverse age cohorts to better understand how digital interventions perform across the lifespan. A further gap in the current evidence concerns patient adherence. None of the included studies systematically evaluated adherence to digital tools, despite several reporting on patient satisfaction. Understanding the drivers and barriers of sustained engagement is essential for real-world implementation and scale-up, as adherence likely mediates the effectiveness of digital interventions. Finally, the scope of this review may be constrained by methodological limitations in the search strategy. Specifically, the exclusion of grey literature and the limited number of databases consulted may have led to the omission of relevant studies, particularly those in early implementation stages or reported in nonindexed formats. So future updates could include grey literature and trial registries to minimize publication bias and broaden coverage.

## 5. Conclusions

Telehealth interventions for individuals with T2DM show promising potential in improving glycemic control and supporting self-management. However, their effectiveness in managing broader cardiovascular risk factors remains less clear due to inconsistent reporting and heterogeneity of intervention designs.

This scoping review adds to the literature by focusing on AI-integrated telehealth tools for the joint management of diabetes and cardiovascular risk. By centering on dual-condition outcomes, our findings offer a novel perspective that extends beyond disease-specific care models and identifies specific evidence gaps that future trials should address.

Future research, particularly involving AI-integrated tools, must address critical implementation challenges such as data quality, clinical validation, regulatory adherence, and equitable access. In addition, future research should prioritize the use of standardized outcome measures, longer follow-up periods, and inclusive methodologies that address equity, user burden, and system-level integration. The incorporation of both patient and provider perspectives will be vital to optimizing digital health tools for integrated, sustainable, and equitable chronic disease management.

## Figures and Tables

**Table 1:** Studies' characteristics.

**First author [ref]**	**Year of publication**	**Country**	**Study design**	**Funding**	**Trial ID**	**Participants (** **n** **)**	**Age (years) (** **m** **e** **a** **n** ± **SD) or (median, IQR)**	**Sex (%)**	**Race/ethnicity (%)**	**Health condition**	**Comorbidities (%)**
Li C [50]	2025	China	Prospective observational study	Nonindustry	NA	Baseline: 199,431 After 3-month follow-up: 118,134	57.3 ± 12.5	Females: 42.8 Males: 57.2	NR	T2DM initiating basal insulin therapy	Hypertension: 28.4 Hyperlipidemia: 16.4 Atrial fibrillation: 0.15 Left ventricular hypertrophy: 0.10

Warren M [48]	2024	United States	Retrospective observational study	Industry	NA	600	67.1 ± 11.5	Females: 47.2 Males: 52.8	Caucasian: 53.8 African American: 37.2 Asian: 0.3 American Indian or Alaskan Native: 0.8 Other: 1.7 Not reported: 6.2 Hispanic or Latino: 0.8 Not Hispanic or Latino: 1.2 Not reported: 98	T2DM using insulin	Hypertension: 66.3 Dyslipidemia: 64.8 Cardiovascular morbidity: 36.0 Coronary artery disease: 23.5 History of stroke: 15.5 Heart failure: 11.0 Peripheral vascular disease: 8.2

De Luca V [52]	2023	Italy	Non-RCT exploratory trial	Nonindustry	NA	Intervention: 100 Control 100	Intervention: 61.1 ± 9.4 Control: 66.5 ± 9.0	Intervention: Females: 17 Males: 83 Control: Females: 30 Males: 70	NR	T2DM	NR

Lallemand A [53]	2023	Belgium	Quantitative quasiexperimental study	Industry nonindustry	NA	66	56.68 ± 13.95	Female: 56.06 Male: 43.94	NR	T2DM	Obesity Classes I–III: 48.49 Overweight: 21.21

Oseran AS [49]	2021	USA	Cluster-randomized matched cohort study	Nonindustry	NCT03542084	Intervention: 130 Control: 130	Intervention: 62.6 ± 11.6 Control: 61.3 ± 11.0	Intervention: Female: 46.9 Male: 53.1 Control: Female: 44.6 Male: 55.4	Intervention: Black: 11.5 Hispanic: 10.8 White: 50.8 Other: 26.9 Control: Black: 10.0 Hispanic: 13.1 White: 53.1 Other: 23.8	T2DM	Intervention: Hyperlipidemia: 94.6 Hypertension: 80.0 Coronary artery disease: 24.6 Chronic kidney disease:19.2 Congestive heart failure: 13.8 Peripheral vascular disease: 11.5 Control: Hyperlipidemia: 94.6 Hypertension:90.8 Coronary artery disease: 18.5 Chronic kidney disease: 16.1 Congestive heart failure: 13.1 Peripheral vascular disease: 10.0

Finkelstein EA [51]	2025	Singapore	RCT	Nonindustry	NCT03800680	UC group: 110 DMP group: 31 DMP+ group: 105	UC group: 54.7 ± 9.77 DMP group: 56.1 ± 8.38 DMP+ group: 54.9 ± 10.05	UC group: Female: 37.3 Male: 62.7 DMP group: Female: 35.5 Male: 64.5 DMP+ group: Female 34.3 Male 65.7	UC group: Chinese: 79.1 Malay: 3.6 Indian and others: 17.3 DMP group: Chinese: 77.4 Malay: 9.7 Indian and others: 12.9 DMP+ group: Chinese: 74.3 Malay: 4.8 Indian and others: 20.9	T2DM	NR

Abbreviations: DMP group, diabetes management program; DMP+ group, DMP, with M-POWER rewards (via M-Power app); IQR, interquartile range; NA, not applicable; NR, not reported; RCT, randomized controlled trial; SD, standard deviation; T2DM, Type 2 diabetes mellitus; UC group, usual care (control).

**Table 2:** Main characteristics of the intervention applied in each study.

**First author [ref]**	**Intervention**					
	**Designation**	**Type**	**Main features**	**Other relevant information**	**Duration (months)**	**Randomization process**
Li C [50]	TRIO	Mobile app (integrated with WeChat official account) and phone-based follow-up	Data-based and artificial intelligence management system	NR	3	NA (observational study)

Warren M [48]	d-Nav technology	Phone app	AI-based autonomous insulin titration Patients use their own glucometer/CGM to measure glucose before each injection d-Nav provides the recommended dose AI assesses the patient's glucose patterns, titrates doses at least weekly, and adjusts doses to balance hyperglycemia/hypoglycemia prevention Recommends dose reduction for hypoglycemia (safety-first) Supports basal-only premix twice a day and basal-bolus (with/without carb counting) regimens Includes clinical support (onboarding training, virtual monitoring, and communication by specialists) Providers can review patient data	FDA cleared (in use: The United States since 2019 and Europe for > 10 years) Covered by most health plans, including Medicare	8.2 ± 3.0	NA (observational study)

De Luca V [52]	ProEmpower solutions: - DM4All - DiaWatch	Mobile app integrated with medical device web interface	Collection of clinical parameters by a patient using a smartphone integrated with medical devices (glucometer, sphygmomanometer scale, smartwatch for heart rate, and step counter) Data automatically sent to a shared care plan accessible by patients and professionals includes info on lifestyle, treatment plans, and disease-related data Professionals can monitor adherence, set goals, and communicate Solutions automatically send suggestions to patients when they deviate from treatment targets Coaching on healthy lifestyle promotion Promoting self-care and continuous monitoring	Control cohort of T2DM patients (*n* = 100) with similar clinical characteristics, followed for a comparable period, receiving usual care in the same centers	8	Randomization was performed at the healthcare center level Each center used a single solution

Lallemand A [53]	Comunicare health application	Mobile app for patients and a dashboard for the healthcare team (pharmacists)	Sections for patient input (“My medication,” “My follow-up,” and “My feelings” transferring data on state of mind, hypoglycemic episodes, blood glucose measurements, medication intake, and physical activity) Therapeutic education information (“My advice”) Appointment list (“My agenda”) Integrated videoconferencing technology Supports the French and Dutch languages	A new configuration of the Comunicare platform specifically tailored to the follow-up of diabetes was created for this project	6	NA (quasiexperimental study)

Oseran AS [49]	Unsolicited endocrinology eConsult system	Electronic consultation (eConsult) system integrated with EHR	HbA1c-triggered eConsult based on EHR data/rules	Control group: Patients of PCPs randomized to the control arm received the usual standard of care PCPs in both arms could still actively request eConsults via the pre-existing program Consulting endocrinologists paid $52/eConsult	6	PCPs were randomly assigned to intervention (*n* = 81) and control (*n* = 80) arms Due to the nature of the intervention, study investigators and consulting endocrinologists were not blinded

Finkelstein EA [51]	GlycoLeap mobile app (core) Fitbit app RxCap/Medisafe medication app M-POWER app	Mobile apps and connected devices (Fitbit activity tracker, glucometer, and digital weighing scale)	Diabetes education Health behavior tracking (blood glucose, weight, diet, and physical activity) Health coaching Medication adherence tracking Rewards for healthy behaviors and outcomes (DMP+ group): M-POWER app for rewards	DMP+ rewards disbursed as rebates for approved outpatient and exercise-related expenditures Maximum annual value of SGD516 (USD378) M-POWER is based on behavioral economic theory	12	Block randomized to one of three arms in a 10:1:10 ratio—UC, DMP, and DMP+ by the project coordinator at Duke-NUS Medical School Three stratification factors used: Usual source of diabetes care (primary care or specialist clinic), gender (male or female), and dichotomized HbA1c at baseline (≤ 9.2% or > 9.2%)

Abbreviations: CGM, continuous glucose monitor; DMP group, diabetes management program; DMP+ group, DMP, with M-POWER rewards (via M-Power app); EHR, electronic health record; FDA, Food and Drug Administration; NA, not applicable; NR, not reported. PCP, primary care physician; T2DM, Type 2 diabetes mellitus; UC group, usual care (control).

**Table 3:** Outcomes of interest at baseline and endpoint, group comparison as presented by the authors of each study, overall improvements regarding management of diabetes, cardiovascular risk, and other relevant outcomes related to the intervention.

**First author (ref)**	**Outcomes of interest**	**Outcomes of interest**	**Statistical significance**	**Overall improvement**	**Other relevant reported outcomes**
	**BASELINE**	**ENDPOINT**			
	**M** **e** **a** **n** ± **SD or median (IQR)**	**M** **e** **a** **n** ± **SD**			
Observational and quasiexperimental studies					
Li C [50]	HbA1c (%): 9.6 ± 2.0 FBG (mmol/L): 9.5 ± 3.3 SBP (mm Hg): 131.8 ± 16.2 DBP (mm Hg): 79.7 ± 10.3 TG (mmol/L): 2.3 ± 2.1 TC (mmol/L): 4.7 ± 1.5 LDL-c (mmol/L): 2.8 ± 1.1	HbA1c (%): 6.89 ± 0.90 (for *n* = 44847 with eligible self-reported HbA1c) FBG (mmol/L): 6.81 ± 1.4 (for *n* = 60365 with eligible self-reported FBG) SBP/DBP/TG/TC/LDL-c: Not reported at the 3-month endpoint for the total cohort	HbA1c (%): −2.59 ± 0.01 (*p* < 0.001) FBG (mmol/L): −2.77 ± 0.01 (*p* < 0.001)	Improved glycemic control (HbA1c and FBG reduction)	Target achievement: HbA1c < 7%: 55.6% FBG < 7.0 mmol/L: 61.3% FBG < 6.1 mmol/L: 29.2% Safety: Hypoglycemia incidence (≤ 3.9 mmol/L): 27.1% Patient satisfaction: 99.6% of the patients felt satisfactory or very satisfactory at Month 3

Warren M [48]	HbA1c (%): 7.9 ± 1.8 Complete cohort: 8.6 ± 2.1 (*n* = 54)	HbA1c (%): 7.2 ± 1.0 Complete cohort: 7.6 ± 1.5	*p* = 0.002 Complete cohort: *p* < 0.0001	NR	Hypoglycemia (< 54 mg/dL): 0.4 ± 0.6 events/month; Severe hypoglycemia: 1.7/100 patient-years Patient satisfaction: High overall score: 3.8 on a 1–4 scale (*n* = 48) Total daily insulin dose: Increased by 60.6% (from 69.3 to 111.3 units); decreased in 21% of patients in the first 3 months due to relatively low glucose levels

Lallemand A [53]	HbA1c (%): 6.49 ± 1.32 SBP (mmHg): 135.49 ± 15.05 DBP (mmHg): 81.86 ± 9.43 LDL-c (mmol/L): 2.01 ± 0.93 HDL-c (mmol/L): 1.39 ± 0.45	HbA1c (%): 6.21 ± 0.83 SBP (mmHg): 135.0 ± 18.92 DBP (mmHg): 79.6 ± 11.63 LDL-c (mmol/L): 1.99 ± 0.97 HDL-c (mmol/L): 1.47 ± 0.49	Endpoint at 6 months (*n* = 46) HbA1c (%): −0.1 ± 0.54 (*p* = 0.37) SBP (mmHg): −2.48 ± 19.22 (*p* = 0.41) DBP (mmHg): −2.25 ± 11.39 (*p* = 0.07) LDL-c (mmol/L): −0.06 ± 0.36 (*p* = 0.35) HDL-c (mmol/L): 0.06 ± 0.24 (*p* = 0.17)	Reduction of body weight and waist circumference	Patient satisfaction: Patients appreciated the contact with the healthcare provider, the individualized follow-up, and support Patients found the application content interesting and useful Health professional satisfaction: Pharmacists felt their usefulness

Experimental studies					
Finkelstein EA [51]	HbA1c (%) UC group: 8.22 ± 0.96 DMP group: 8.25 ± 1.23 DMP+ group: 7.93 ± 0.82 SBP (mmHg) UC group: 121.6 ± 14.0 DM group: 122.7 ± 10.4 DMP+ group: 122.6 ± 13.4 DBP (mmHg) UC group: 75.6 ± 8.5 DMP group: 77.3 ± 8.6 DMP+ group: 76.7 ± 8.8	HbA1c (%) UC group: −0.13 DMP+ group: −0.51 SBP (mmHg) UC group: −2.47 DMP+ group: −3.72 DBP (mmHg) UC group: −1.80 DMP+ group: −2.48	HbA1c *p* = 0.015 SBP *p* = 0.384 DBP *p* = 0.434	DMP+ participants had greater self-monitoring: Weight: MD = 0.49; 95% CI, −0.03, 1.02) (*p* = 0.065) Blood glucose per week: MD = 0.85; 95% CI, 0.33, 1.36) (*p* = 0.001) Diabetes self-management questionnaire: MD = +0.31 (95% CI, 0.08, 0.53), range 0–10, higher score indicates more effective self-management	Quality of life (EQ-5D-5L utility index): No significant difference (MD = 0.00) Sleep quality (PSQI total score): DMP+ reported poorer sleep quality (MD = 0.71, *p* = 0.037) Work productivity (WPAI:SHP % overall work impairment): DMP+ reported greater work impairment (MD = 5.53, *p* = 0.068)

Oseran AS [49]	Intervention: HbA1c (%): 9.5 ± 0.9 SBP (mmHg): 130.2 ± 18.9 TC (mg/dL): 166.3 ± 40.2 LDL-c (mg/dL): 90.5 ± 35.5 Control: HbA1c (mmol/mol): 9.4 ± 0.6 SBP (mmHg): 132.2 ± 16.6 TC (mg/dL): 164.7 ± 39.1 LDL-c (mg/dL): 81.4 ± 32.5	Primary outcome: HbA1c (Mean change ± SD) Intervention: 6 months: −0.83 ± 1.41 12 months: −0.75 ± 1.48 18 months: −0.85 ± 1.42 Control: 6 months: −0.72 ± 1.29 12 months: −0.68 ± 1.39 18 months: −0.88 ± 1.42	6 months: *p* = 0.55 12 months: *p* = 0.74 18 months: *p* = 0.91	NR	Prescribing of medications (GLP-1 RA or SGLT2i): +19.3% in intervention vs. +6.9% in control (*p* = 0.003) In-person endocrinology visits: 13 in intervention arm vs. 29 in control arm (*p* = 0.012) PCP implementation of eConsult recommendations: 38% overall (28% for new medication addition and 53% for dose adjustment)

De Luca V [52]	Intervention: HbA1c (%): 6.99 ± 0.95 (*n* = 84) SBP (mmHg): 135.6 ± 18.0 (*n* = 92) DBP (mmHg): 80.5 ± 9.8 (*n* = 92) TG (mg/dL): 139.9 ± 81.3 (*n* = 45) TC (mg/dL): 179.9 ± 49.3 (*n* = 46) LDL-c (mg/dL): 109.1 ± 43.2 (*n* = 39) HDL-c (mg/dL): 43.0 ± 9.9 (*n* = 48) Control: HbA1c (%): 7.04 ± 0.93 (*n* = 100) SBP (mmHg): 131.4 ± 16.9 (*n* = 39) DBP (mmHg): 78.2 ± 7.7 (*n* = 39) TG (mg/dL): 140.1 ± 56.8 (*n* = 55) TC (mg/dL): 166.4 ± 38.1 (*n* = 59) LDL-c (mg/dL): 91.1 ± 31.7 (*n* = 52) HDL-c (mg/dL): 46.2 ± 12.6 (*n* = 59)	Intervention: HbA1c (%): 6.76 ± 0.87 (*n* = 84)⁣^∗^ SBP (mmHg): 126.1 ± 14.5 (*n* = 92)⁣^∗^ DBP (mmHg): 76.2 ± 8.9 (*n* = 92)⁣^∗^ TG (mg/dL): 133.4 ± 81.1 (*n* = 45) TC (mg/dL): 158.1 ± 37.9 (*n* = 46)⁣^∗^ LDL-c (mg/dL): 86.0 ± 32.1 (*n* = 39)⁣^∗^ HDL-c (mg/dL): 44.4 ± 10.0 (*n* = 48) Control: HbA1c (%): 6.90 ± 0.87 (*n* = 100)⁣^∗^ SBP (mmHg): 132.7 ± 15.0 (*n* = 39) DBP (mmHg): 79.9 ± 7.6 (*n* = 39) TG (mg/dL): 147.0 ± 54.9 (*n* = 55) TC (mg/dL): 164.5 ± 35.4 (*n* = 59) LDL-c (mg/dL): 88.4 ± 31.9 (*n* = 52) HDL-c (mg/dL): 45.2 ± 12.4 (*n* = 59)	Between cohorts (*p* value): HbA1c: *p* = 0.555 SBP: *p* < 0.001 DBP: *p* < 0.001 TG: *p* = 0.252 TC: *p* = 0.003 LDL-c: *p* = 0.002 HDL-c: *p* = 0.095	Increased self-management of diabetes (qualitative report by participants). Improvement of metabolic and other outcomes (body weight) relevant to CV risk	Patient satisfaction and usability were reported in a different reference mentioned by the authors^a^

^a^De Luca V., Bozzetto L., Giglio C., Tramontano G., Chiatti C., Gonidis F., et al., (2021). Satisfaction, self-management and usability: assessment of two novel IT solutions for type 2 diabetes patients' empowerment. Proceedings of the 7^th^ International Conference on Information and Communication Technologies for Ageing Well and e-health.

⁣^∗^Statistically significant compared with baseline.

## Data Availability

Data sharing is not applicable to this article as no datasets were generated or analyzed during the current study.

## Supporting Information

Supporting Information 1 Supporting Information S1: PRISMA 2020 Checklist.

Supporting Information 2 Supporting Information S2: Search string used in each database and the respective number of retrieved articles.
