# Supplementary material for: Telehealth for Integrated Cardiovascular and Diabetes Management: A Scoping Review
Source: J Diabetes Res. 2025 Dec 2;2025:1093671. doi: 10.1155/jdr/1093671 (PMC12688643; doi:10.1155/jdr/1093671)
Supplement: Supporting Information 2 — Supporting Information S2: Search string used in each database and the respective number of retrieved articles. [file 1093671.f2.docx]

| **Database Hits** | |
| --- | --- |
| **PubMed** | 578 |
| (Telemedicine[MeSH Terms] OR Remote Consultation[MeSH Terms] OR Monitoring, Ambulatory[MeSH Terms] OR telehealth[Title/Abstract] OR telemedicine[Title/Abstract] OR "remote monitoring"[Title/Abstract] OR "remote consultation"[Title/Abstract] OR "digital health"[Title/Abstract] OR mHealth[Title/Abstract] OR eHealth[Title/Abstract] OR "virtual health"[Title/Abstract] OR telecare[Title/Abstract] OR telemonitoring[Title/Abstract] OR teleconsultation[Title/Abstract] OR "mobile health"[Title/Abstract] OR "electronic health"[Title/Abstract] OR "artificial intelligence"[Title/Abstract] OR AI[Title/Abstract] OR "machine learning"[Title/Abstract] OR "deep learning"[Title/Abstract] OR "predictive analytics"[Title/Abstract] OR algorithm[Title/Abstract] OR "automated system"[Title/Abstract] OR "smart health"[Title/Abstract] OR "intelligent monitoring"[Title/Abstract] OR chatbot[Title/Abstract] OR "virtual assistant"[Title/Abstract]) **AND** ("Cardiovascular Diseases"[MeSH Terms] OR "Heart Diseases"[MeSH Terms] OR "Vascular Diseases"[MeSH Terms] OR "Coronary Disease"[MeSH Terms] OR "Cerebrovascular Disorders"[MeSH Terms] OR "Hypertension"[MeSH Terms] OR "Blood Pressure"[MeSH Terms] OR "Dyslipidemias"[MeSH Terms] OR "Cholesterol, LDL"[MeSH Terms] OR "Triglycerides"[MeSH Terms] OR "Heart Failure"[MeSH Terms] OR "cardiovascular disease"[Title/Abstract] OR "heart disease"[Title/Abstract] OR hypertension[Title/Abstract] OR dyslipidemia[Title/Abstract] OR "LDL cholesterol"[Title/Abstract] OR triglycerides[Title/Abstract] OR "systolic blood pressure"[Title/Abstract] OR "diastolic blood pressure"[Title/Abstract] OR "cardiac disease"[Title/Abstract] OR "Heart Failure"[Title/Abstract] OR "vascular disease"[Title/Abstract] OR "coronary artery disease"[Title/Abstract] OR stroke[Title/Abstract] OR "peripheral artery disease"[Title/Abstract]) **AND** ("Diabetes Mellitus"[MeSH Terms] OR "Diabetes Mellitus, Type 2"[MeSH Terms] OR "Diabetes Mellitus, Type 1"[MeSH Terms] OR "Blood Glucose"[MeSH Terms] OR "Self-Management"[MeSH Terms] OR "diabetes mellitus"[Title/Abstract] OR "type 2 diabetes"[Title/Abstract] OR "type 1 diabetes"[Title/Abstract] OR "glyc* control"[Title/Abstract] OR "HbA1c"[Title/Abstract] OR "A1c"[Title/Abstract] OR "glyc* hemoglobin"[Title/Abstract] OR "fasting glucose"[Title/Abstract] OR "blood glucose"[Title/Abstract] OR "diabetes management"[Title/Abstract] OR "diabetes care"[Title/Abstract] OR "diabetes self-management"[Title/Abstract] OR "diabetic complications"[Title/Abstract]) **AND** ("Integrated Care"[MeSH Terms] OR "Comorbidity"[MeSH Terms] OR "Disease Management"[MeSH Terms] OR "Patient-Centered Care"[MeSH Terms] OR "integrated care"[Title/Abstract] OR "coordinated care"[Title/Abstract] OR "collaborative care"[Title/Abstract] OR "concurrent management"[Title/Abstract] OR multimorbidity[Title/Abstract] OR comorbidity[Title/Abstract] OR "co-management"[Title/Abstract] OR "combined management"[Title/Abstract] OR "dual management"[Title/Abstract] OR "combined care"[Title/Abstract] OR "patient-centered care"[Title/Abstract]) |  |
| **Web of Science** | 584 |
| (TS=(Telemedicine) OR TS=("Remote Consultation") OR TS=("Monitoring, Ambulatory") OR TS=(telehealth) OR TS=(telemedicine) OR TS=("remote monitoring") OR TS=("remote consultation") OR TS=("digital health") OR TS=(mHealth) OR TS=(eHealth) OR TS=("virtual health") OR TS=(telecare) OR TS=(telemonitoring) OR TS=(teleconsultation) OR TS=("mobile health") OR TS=("electronic health") OR TS=("artificial intelligence") OR TS=(AI) OR TS=("machine learning") OR TS=("deep learning") OR TS=("predictive analytics") OR TS=(algorithm) OR TS=("automated system") OR TS=("smart health") OR TS=("intelligent monitoring") OR TS=(chatbot) OR TS=("virtual assistant")) **AND** (TS=("Cardiovascular Diseases") OR TS=("Heart Diseases") OR TS=("Vascular Diseases") OR TS=("Coronary Disease") OR TS=("Cerebrovascular Disorders") OR TS=(Hypertension) OR TS=("Blood Pressure") OR TS=(Dyslipidemias) OR TS=("Cholesterol, LDL") OR TS=(Triglycerides) OR TS=("Heart Failure") OR TS=("cardiovascular disease") OR TS=("heart disease") OR TS=(hypertension) OR TS=(dyslipidemia) OR TS=("LDL cholesterol") OR TS=(triglycerides) OR TS=("systolic blood pressure") OR TS=("diastolic blood pressure") OR TS=("cardiac disease") OR TS=("Heart Failure") OR TS=("vascular disease") OR TS=("coronary artery disease") OR TS=(stroke) OR TS=("peripheral artery disease")) **AND** (TS=("Diabetes Mellitus") OR TS=("Diabetes Mellitus, Type 2") OR TS=("Diabetes Mellitus, Type 1") OR TS=("Blood Glucose") OR TS=("Self-Management") OR TS=("diabetes mellitus") OR TS=("type 2 diabetes") OR TS=("type 1 diabetes") OR TS=("glyc* control") OR TS=(HbA1c) OR TS=(A1c) OR TS=("glyc* hemoglobin") OR TS=("fasting glucose") OR TS=("blood glucose") OR TS=("diabetes management") OR TS=("diabetes care") OR TS=("diabetes self-management") OR TS=("diabetic complications")) **AND** (TS=("Integrated Care") OR TS=(Comorbidity) OR TS=("Disease Management") OR TS=("Patient-Centered Care") OR TS=("integrated care") OR TS=("coordinated care") OR TS=("collaborative care") OR TS=("concurrent management") OR TS=(multimorbidity) OR TS=(comorbidity) OR TS=("co-management") OR TS=("combined management") OR TS=("dual management") OR TS=("combined care") OR TS=("patient-centered care")) |  |
| **Scopus** | 2572 |
| (INDEXTERMS(Telemedicine) OR INDEXTERMS("Remote Consultation") OR INDEXTERMS("Monitoring, Ambulatory") OR TITLE-ABS(telehealth) OR TITLE-ABS(telemedicine) OR TITLE-ABS("remote monitoring") OR TITLE-ABS("remote consultation") OR TITLE-ABS("digital health") OR TITLE-ABS(mHealth) OR TITLE-ABS(eHealth) OR TITLE-ABS("virtual health") OR TITLE-ABS(telecare) OR TITLE-ABS(telemonitoring) OR TITLE-ABS(teleconsultation) OR TITLE-ABS("mobile health") OR TITLE-ABS("electronic health") OR TITLE-ABS("artificial intelligence") OR TITLE-ABS(AI) OR TITLE-ABS("machine learning") OR TITLE-ABS("deep learning") OR TITLE-ABS("predictive analytics") OR TITLE-ABS(algorithm) OR TITLE-ABS("automated system") OR TITLE-ABS("smart health") OR TITLE-ABS("intelligent monitoring") OR TITLE-ABS(chatbot) OR TITLE-ABS("virtual assistant")) **AND** (INDEXTERMS("Cardiovascular Diseases") OR INDEXTERMS("Heart Diseases") OR INDEXTERMS("Vascular Diseases") OR INDEXTERMS("Coronary Disease") OR INDEXTERMS("Cerebrovascular Disorders") OR INDEXTERMS(Hypertension) OR INDEXTERMS("Blood Pressure") OR INDEXTERMS(Dyslipidemias) OR INDEXTERMS("Cholesterol, LDL") OR INDEXTERMS(Triglycerides) OR INDEXTERMS("Heart Failure") OR TITLE-ABS("cardiovascular disease") OR TITLE-ABS("heart disease") OR TITLE-ABS(hypertension) OR TITLE-ABS(dyslipidemia) OR TITLE-ABS("LDL cholesterol") OR TITLE-ABS(triglycerides) OR TITLE-ABS("systolic blood pressure") OR TITLE-ABS("diastolic blood pressure") OR TITLE-ABS("cardiac disease") OR TITLE-ABS("Heart Failure") OR TITLE-ABS("vascular disease") OR TITLE-ABS("coronary artery disease") OR TITLE-ABS(stroke) OR TITLE-ABS("peripheral artery disease")) **AND** (INDEXTERMS("Diabetes Mellitus") OR INDEXTERMS("Diabetes Mellitus, Type 2") OR INDEXTERMS("Diabetes Mellitus, Type 1") OR INDEXTERMS("Blood Glucose") OR INDEXTERMS(Self-Management) OR TITLE-ABS("diabetes mellitus") OR TITLE-ABS("type 2 diabetes") OR TITLE-ABS("type 1 diabetes") OR TITLE-ABS("glyc* control") OR TITLE-ABS(HbA1c) OR TITLE-ABS(A1c) OR TITLE-ABS("glyc* hemoglobin") OR TITLE-ABS("fasting glucose") OR TITLE-ABS("blood glucose") OR TITLE-ABS("diabetes management") OR TITLE-ABS("diabetes care") OR TITLE-ABS("diabetes self-management") OR TITLE-ABS("diabetic complications")) AND (INDEXTERMS("Integrated Care") OR INDEXTERMS(Comorbidity) OR INDEXTERMS("Disease Management") OR INDEXTERMS("Patient-Centered Care") OR TITLE-ABS("integrated care") OR TITLE-ABS("coordinated care") OR TITLE-ABS("collaborative care") OR TITLE-ABS("concurrent management") OR TITLE-ABS(multimorbidity) OR TITLE-ABS(comorbidity) OR TITLE-ABS(co-management) OR TITLE-ABS("combined management") OR TITLE-ABS("dual management") OR TITLE-ABS("combined care") OR TITLE-ABS("patient-centered care")) |  |
